# Reversible Solubility Switching of a Polymer Triggered by Visible‐Light Responsive Azobenzene Photochromism with Negligible Thermal Relaxation

**DOI:** 10.1002/marc.202400419

**Published:** 2024-08-08

**Authors:** Takeshi Ueki, Yuna Osaka, Kenta Homma, Shota Yamamoto, Aya Saruwatari, Hongxin Wang, Masao Kamimura, Jun Nakanishi

**Affiliations:** ^1^ Research Center for Macromolecules and Biomaterials National Institute of Materials and Science 1‐1 Namiki Tsukuba Ibaraki 305‐0044 Japan; ^2^ Graduate School of Life Science Hokkaido University Kita 10, Nishi 8, Kita‐ku Sapporo Hokkaido 060–0810 Japan; ^3^ Graduate School of Advanced Engineering Tokyo University of Science 6‐3‐1 Niijuku, Katsushika‐ku Tokyo 125–8585 Japan; ^4^ Graduate School of Advanced Science and Engineering Waseda University 3‐4‐1 Okubo Shinjuku‐ku Tokyo 169–8555 Japan; ^5^ Present address: Department of Applied Chemistry Graduate School of Engineering Osaka University 2‐1 Yamadaoka Suita Osaka 565–0871 Japan; ^6^ Present address: Center for Future Innovation (CFi), Graduate School of Engineering Osaka University 2‐1 Yamadaoka Suita Osaka 565–0871 Japan

**Keywords:** azobenzene, gels, LCST, mechanobiology, phase transition

## Abstract

This study reports the reversible solubility switching of a polymer triggered by non‐phototoxic visible light. A photochromic polymerizable azobenzene monomer with four methoxy groups at the *ortho*‐position (mAzoA) was synthesized, exhibiting reversible photoisomerization between *trans*‐ and *cis*‐states using green (546 nm) and blue light (436 nm). Free radical copolymerization of hydrophilic dimethylacrylamide (DMAAm) with mAzoA produced a light‐responsive random copolymer (P(mAzoA‐*r*‐DMAAm)) that shows a reversible photochromic reaction to visible light. Optimizing mAzoA content resulted in P(mAzoA_10.7_‐*r*‐DMAAm)_3.0 kDa_ exhibiting LCST‐type phase separation in PBS (pH 7.4) with *trans*‐ and *cis*‐states at 39.2 °C and 32.9 °C, respectively. The bistable temperature range of 6.3 °C covers 37 °C, suitable for mammalian cell culture. Reversible solubility changes were demonstrated under alternating green and blue light at 37 °C. 1H NMR indicated significant retardation of thermal relaxation from *cis*‐ to *trans*‐states, preventing undesired thermal mechanical degradation. Madin Darby Canine Kidney (MDCK) cells adhered to the P(mAzoA‐*r*‐DMAAm) hydrogel, confirming its non‐cytotoxicity and potential for biocompatible interfaces. This principle is useful for developing hydrogels that can reversibly stimulate cells mechanically or chemically in response to visible light.

## Graphical abstract

A stimuli‐responsive polymer is reported that possesses azobenzene with four methoxy groups at the ortho‐position. This polymer exhibits reversible solubility changes induced by visible light. The discovery contributes to the realization of mechanobiological scaffolds that are free from concerns about phototoxicity. Furthermore, this material does not suffer from the undesired changes in material properties due to thermal relaxation.

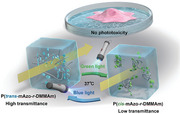

## 1 Introduction

Cells alter their phenotypes (e.g., spreading, proliferation, migration, and differentiation) in response not only to biochemical but also to mechanical factors of the surrounding environment.^[^ ^1^ ^]^ Synthetic hydrogel scaffolds that mimic mechanical property changes in vivo have been actively proposed.^[^ ^2^ ^]^ Recently, materials that achieve bidirectional and reversible changes in mechanical properties have been reported. Pioneering works have developed hydrogels such as pH‐responsive triblock copolymer gels^[^ ^3^ ^]^ and cyclodextrins (CDs) involving supramolecular gels.^[^ ^4^ ^]^ These studies have revealed impressive results suggesting that periodic mechanical stimulation may maintain the undifferentiation of stem cells^[^ ^3b^ ^]^ or induce periodic changes in cell spreading area.^[^ ^4b^ ^]^ However, the use of chemical stimuli inducers to cause mechanical forces limits the applicability of the material, because they must be added directly to the cell culture environment, which may cause cellular metabolic changes and complicate the experimental system. Anseth et al. reported a two‐way modulus‐changeable hydrogel scaffold induced by facile photo‐triggers.^[^ ^5^ ^]^ To achieve reversible mechanical property changes, they combined the photocleavage reaction of nitrobenzene with subsequent photopolymerization of methacrylate pre‐polymer in a cell scaffold. However, in principle, this system can induce only one cycle of modulus change, which may limit the application of repetitive mechanical stimulation to cells. Well‐designed bidirectional systems that incorporate conformational changes in enzyme proteins at cross‐linking have also been reported.^[^ ^6^ ^]^ However, these systems are problematic because of the risk of accidental contamination brought from insufficiently purified biopolymers and their high cost.

Azobenzene is a typical T‐type (thermal type) photochromic molecule that undergoes photoisomerization with excellent cycle durability.^[^ ^7^ ^]^ If the two photoisomerization states (*trans*/*cis*) of azobenzene can be incorporated into the material, straightforward cell scaffold^[^ ^8^ ^]^ materials that can switch their mechanical (and/or chemical) properties repeatedly by simply switching the irradiation wavelengths can be realized. However, two problems are associated with the use of azobenzene as a cell scaffold. First problem is the phototoxicity of UV light that induces a π‐π* transition during photoisomerization from *trans*‐ to *cis*‐azobenzene. The second problem is the thermal instability of *cis*‐azobenzene. Because *cis*‐azobenzene is thermally metastable, it gradually returns to the most stable *trans*‐azobenzene unless UV light is continuously illuminated. Hence, this problem leads to an undesirable change of the mechanical properties from *cis*‐ to *trans*‐azobenzene‐containing materials. If a mechanical property switching system that is photoisomerizable only by visible light irradiation without phototoxicity and yet displays very slow thermal relaxation can be developed, it will be possible to realize a cell scaffold that will contribute to more sophisticated mechanobiology.

**Figure 1:**
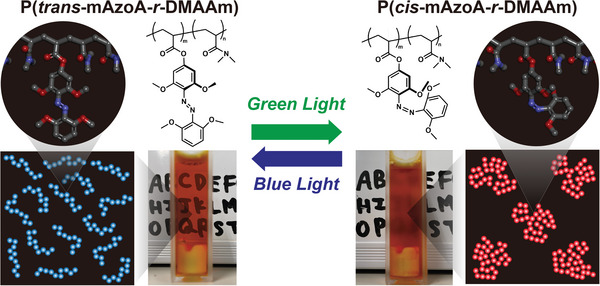
Conceptual illustration of phase transition of P(mAzoA‐*r*‐DMAAm) induced by visible light illumination. (Left) Under blue light illumination, the photoisomerization state of mAzoA along with the polymer becomes *trans*‐rich, giving a transparent polymer solution with coiled conformation. (Right) When irradiated with green light, the photoisomerization state of mAzoA along with the polymer becomes *cis*‐rich, resulting in a globule state of the polymer chain and phase separation.

Herein, we describe a light‐responsive linear polymer (P(mAzoA‐*r*‐DMAAm)) containing an azobenzene derivative with four methoxy groups at the ortho‐positions (**Figure** **1**). The polymer markedly changes its solubility in response to visible light at 37 °C, a typical mammalian cell culture temperature. Various types of visible‐light responsive azobenzenes have already been designed.^[^ ^9^ ^]^ In this study, we selected methoxyazobenzene as the chromophore because it is more hydrophilic by the introduction of 4 methoxy groups and has already been attempted in hydrogel.^[^ ^10^ ^]^ When oriented toward bio‐related applications, methoxy azobenzene has been pointed out to have low resistance against the reduction of glutathione,^[^ ^7^, ^11^ ^]^ which is present inside cells at ≈10 mM. But this would not be a problem in the present study where it is used as a cell scaffold material outside of the cells. Interestingly, P(mAzoA‐*r*‐DMAAm) undergoes extremely slow thermal relaxation from the *cis*‐ to *trans*‐type. It is able to potentially produce two thermally stable states upon photo stimulation alone, responding similarly to P‐type (photochemical type) photochromic molecules, such as diarylethene, containing polymers.^[^ ^12^ ^]^ Furthermore, we prepared a P(mAzoA‐*r*‐DMAAm)‐based hydrogel cell scaffold material and cultured Madin‐Darby Canine Kidney (MDCK) cells at the interface. It was found that the hydrogel with mAzoA provided cell adhesion, spreading, and cell viability against visible light photo illumination.

## 2 Results and Discussion

Figure S6 (Supporting Information) shows the change in absorption spectra of mAzoA monomer (0.03 w/v% in dimethyl sulfoxide) by visible light: irradiation of *trans*‐rich mAzoA monomer with green light at 546 nm (6.6 mW cm^−2^) decreases the absorption based on the π‐π* transition of the *trans*‐form near 280 nm, and the absorption based on the n‐π* transition that appears ≈470 nm blue‐shifts by 36 nm. Most azobenzene compounds, except for certain bridged azobenzene derivatives,^[^ ^13^ ^]^ have almost the same n‐π* absorption wavelength for both the *cis*‐ and *trans*‐forms; however, mAzoA is unique in that the n‐π* absorption band is different. Woolley et al. reported that the n‐π* transition of the *cis*‐form of 2,2′,6,6′‐tetramethoxy‐4,4′‐diamidoazobenzene, the parent compound of mAzoA, is located at 460 nm, whereas that of the *trans*‐form of the same compound is at 520 nm.^[^ ^14^ ^]^ Thus, there is as large as a 60 nm separation of the n‐π* transition band between the *cis*‐ and *trans*‐form. This is explained by the interaction between the methoxy group and the lone pair of electrons on the nitrogen in *trans*‐2,2′,6,6′‐tetramethoxy‐4,4′‐diamidoazobenzene.^[^ ^14^ ^]^ In contrast to the highly twisted *cis*‐form, which has four bulky methoxy groups in the ortho positions, the *trans*‐form has an electron‐donating methoxy group near the lone pair of electrons on the nitrogen. From this structural reason, it is believed that the highest occupied molecular orbital (HOMO) level of the *trans*‐form increases because of the instability of the HOMO of the *trans*‐form, resulting in a significant red shift in the n‐π* absorption band of the *trans*‐form compared to that of the *cis*‐form. When mAzoA in the *cis*‐form was irradiated at 436 nm, the absorption peak based on the n‐π* transition was red‐shifted, and the absorption based on the π‐π* transition of the *trans*‐form recovered to the original intensity. The ^1^H NMR results also confirmed the reversible photoisomerization from the *cis*‐ to *trans*‐form by visible light irradiation (Figure S7, Supporting Information). The photoisomerization ability of mAzoA was maintained after polymerization: irradiation of P(mAzoA‐*r*‐DMAAm) with green light (546 nm) decreased the ratio of the *trans*‐form in the polymer to 8%, and irradiation with blue light (436 nm) recovered the ratio to 88%. The isomerization ratio of the mAzoA groups in the polymer changes to cyclic when irradiated alternately with green (546 nm) and blue light (436 nm) (Figures S8 and S9, Supporting Information).

**Figure 2:**
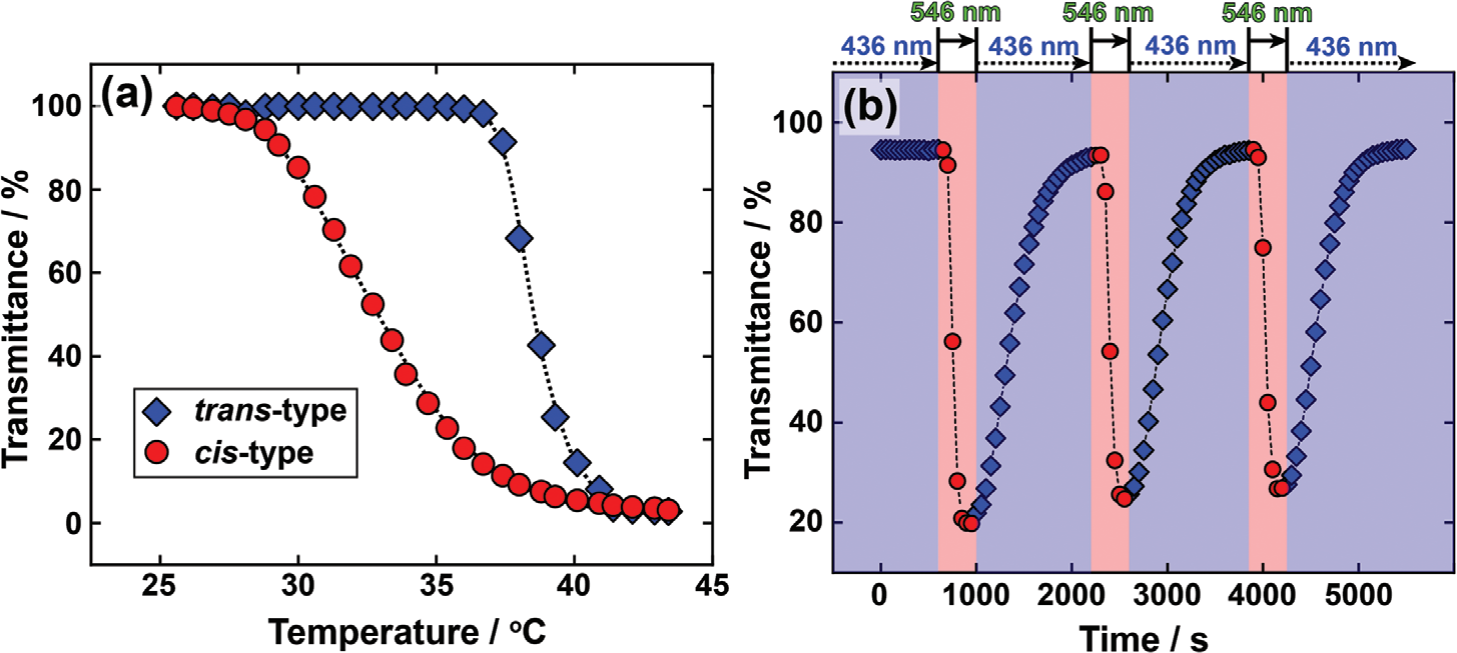
a) Temperature dependence of transmittance at 700 nm for 0.5 wt% P(*trans*‐mAzoA_10.7_‐*r*‐DMAAm)_3.0kDa_ (blue diamond) and for 0.5 wt% P(*cis*‐mAzoA_10.7_‐*r*‐DMAAm)_3.0kDa_ (red circle) in PBS (pH 7.4) solution. The transmittance of 100% indicates that the solution is a single‐phase (transparent), while that of 0% indicates that it is phase‐separated (turbid). Scan rate: 1 °C min^‐1^. b) Cyclic solubility changes between transparent and turbid of P(mAzoA_10.7_‐*r*‐DMAAm)_3.0kDa_ at 37 °C by alternative switching between blue and green light.

The temperature and light sensitivities of P(mAzoA‐*r*‐DMAAm) were evaluated. **Figure** **2**a shows LCST‐type phase transition of 0.5 wt% P(mAzoA_10.7_‐*r*‐DMAAm)_3.0 kDa_ in PBS solution. The subscript numbers indicated after mAzoA and in parentheses express the composition of mAzoA and the number average molecular weight (*M*_n_) of P(mAzoA‐*r*‐DMAAm), respectively. The LCST‐type transition temperature varied depending on the photoisomerization state of mAzoA. *T*_c_s of *trans*‐ and *cis*‐type were 39.2 and 32.9 °C, respectively. Thus, there is a broad enough bistable temperature range of 6.3 °C. This suggests that the solubility of the polymers can be controlled by visible light illumination in the bistable temperature region. In other words, at the typical mammalian cell culture temperature of 37 °C, when the mAzoA group in the polymer chain is *trans* (P(*trans*‐mAzoA_10.7_‐*r*‐DMAAm)_3.0 kDa_) under blue light illumination, the polymer becomes hydrophilic and remains in a soluble state. Conversely, green light produces a *cis*‐type polymer (P(*cis*‐mAzoA_10.7_‐*r*‐DMAAm)_3.0 kDa_) which is eventually insoluble at a constant temperature and phase‐separated. Thus, we attempted to demonstrate the reversible solubility changes of P(mAzoA_10.7_‐*r*‐DMAAm)_3.0 kDa_ induced only by visible light switching at 37 °C (Figure 2b). The transmittance data plotted in the blue region is obtained under 436 nm blue light, whereas the data in the red region is obtained under 546 nm green light. It is clear that P(mAzoA_10.7_‐*r*‐DMAAm)_3.0 kDa_ can repeatedly change its solubility upon switching of visible light. The photo‐induced turbidity change of the polymer (change in the affinity between the polymer and solvent) could be repeatedly induced at least three times. As previously reported, the transmittance lowering process by light irradiation occurs quickly as the polymers undergo a coil‐globule transition in a transparent solution in which light stimuli are effectively absorbed into the azobenzene chromophore.^[^ ^15^ ^]^ The redissolving process of the polymers accompanying the increase in transmittance occurs slowly. This was caused by the light scattering of the polymer aggregates. The transmittance recovery process promotes the irradiation of polymer aggregates with sizes ranging from several hundred nanometers to submicrons. Therefore, light is scattered at the polymer aggregate interface and is not able to penetrate inside the solution effectively. Resulting in a slow dissolution process. PDMAAm (a homopolymer of DMAAm) is hydrophilic and exhibits no thermo‐sensitivity at normal pressure. However, certain random copolymers based on hydrophilic monomers (DMAAm, hydroxyethyl acrylamide, and hydroxyethyl acrylate, etc.) combined with hydrophobic monomers (4‐phenylazophenyl acrylate (AzoA), 4‐phenylazophenyl acrylamide, *tert*‐butyl acrylate (acrylamide), and methyl acrylate, etc.) are known to provide LCST‐type thermo‐sensitivity.^[^ ^16^ ^]^ Because the P(mAzoA‐*r*‐DMAAm) is also a random copolymer of hydrophilic DMAAm and hydrophobic mAzoA, it is reasonable that P(mAzoA‐*r*‐DMAAm) exhibits LCST‐type phase transition.

With some exceptions,^[^ ^17^ ^]^ the LCST‐type phase transition temperatures of azobenzene‐containing polymers in general are observed at low temperatures for the *trans*‐type and at high temperatures for the *cis*‐type.^[^ ^18^ ^]^ The order of phase transition temperatures has been explained by the polarity of azobenzene.^[^ ^7^, ^18^ ^]^ Whereas Figure 2a indicates a higher temperature LCST‐type transition in *trans*‐type polymer and a lower temperature transition in the *cis*‐type polymer, meaning that the trend is the opposite of the generally observed phenomena. Regarding the reversal of the phase transition temperature with respect to the polarity of azobenzene, we have reported that not only the polarity but also the molecular weight of the polymer has a strong influence on *T*_c_.^[^ ^19^ ^]^ In the LCST‐type phase transition of a random copolymer of AzoA with DMAAm (P(AzoA‐*r*‐DMAAm)), for molecular weights above 36 kDa, the order of the phase transition temperature is such general that the higher polar *cis*‐type polymer shows a high‐temperature phase transition and the lower‐polarity *trans*‐type shows a low‐temperature phase transition. However, when the molecular weight was reduced to 16 kDa, the difference in the phase‐transition temperature according to the photoisomerization state disappeared. For the molecular weight of less than 10 kDa, the phase transition temperature of the *trans*‐type is finally higher than that of the *cis*‐type.^[^ ^19^ ^]^ The molecular weight of P(mAzoA‐*r*‐DMAAm) shown in Figure 2a is as low as 3.0 kDa, which is in the molecular weight region where the phase transition temperature exhibits reversal, as expected from previous reports.^[^ ^19^ ^]^ The counterintuitive phase transition temperature order of P(mAzoA_10.7_‐*r*‐DMAAm)_3.0 kDa_ was probably due to the molecular weight effect. Discussion of the effect of [mAzoA] and molecular weight of polymers (Figure S12, Supporting Information) on the LCST‐type phase transition temperature in this system is available on the supporting information.

**Figure 3:**
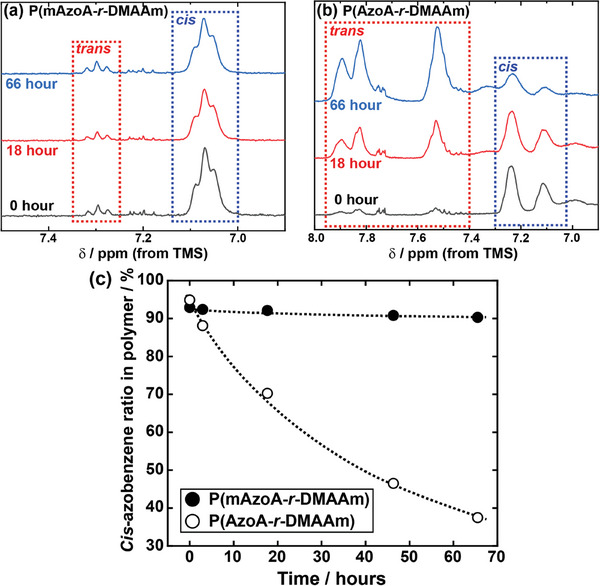
Change in ^1^H NMR spectra at 0, 18, and 66 h during thermal relaxation process from *cis*‐ to *trans*‐type polymer of a) P(mAzoA‐*r*‐DMAAm) and b) P(AzoA‐*r*‐DMAAm) incubated at 37 °C. Before the measurements, P(mAzoA‐*r*‐DMAAm) and P(AzoA‐*r*‐DMAAm) were irradiated with 546 nm and 365 nm light, respectively, to reach each photo‐stationary *cis*‐type polymer. c) Relationship between *cis*‐azobenzene ratio in P(mAzoA‐*r*‐DMAAm) and P(AzoA‐*r*‐DMAAm). The *cis*‐photoisomerization state was estimated from the corresponding integral intensity area of ^1^H NMR spectra.

We also found that the thermal relaxation from the *cis*‐ to the *trans*‐type is remarkably retarded in P(mAzoA‐*r*‐DMAAm) compared to conventional azobenzene‐containing polymers. Thermal relaxation from *cis*‐ to *trans*‐type has been a serious drawback for azobenzene‐containing polymer materials because it causes undesired material aging without a trigger of light illumination. **Figure** **3**a measures the time course of the thermal relaxation process of P(mAzoA‐*r*‐DMAAm) at 37 °C evaluated using ^1^H NMR. For comparison, ^1^H NMR spectra of the thermal relaxation of P(AzoA‐*r*‐DMAAm) with the same thermal history are also shown (Figure 3b). Figure 3c summarizes the decay of the ratio of *cis*‐type azobenzene estimated from the ^1^H NMR in each polymer. P(mAzoA‐*r*‐DMAAm) retained a 90.3% *cis*‐type ratio comparable to the original ratio of 92.9%, even after 66 h of thermal relaxation at 37 °C. This was in sharp contrast to the case of P(AzoA‐*r*‐DMAAm) which kept only a 37.5% *cis*‐type ratio under the same thermal history. Woolley et al. reported that the introduction of methoxy groups into azobenzene not only imparts visible light sensitivity but also slows down the thermal relaxation rate from *cis*‐ to *trans*‐form in low‐molecular‐weight compound systems.^[^ ^14^ ^]^ Here, we have experimentally ensured the remarkable thermal stability of *cis*‐type methoxy‐incorporated azobenzene even after polymerization. The thermal relaxation kinetics from *cis* to *trans* are governed by the potential energy of the ground state of the metastable *cis*‐form and by that of the transition state in the thermal reaction from the *cis*‐ to the *trans*‐form. The introduction of a methoxy group at the ortho‐position of azobenzene was expected to make the ground state of the *cis*‐form more stable, the transition state more unstable, or both, resulting in a slower thermal relaxation rate. A better understanding of the potential energy in each state would explain thermal stability; however, this is a topic for future research.

**Figure 4:**
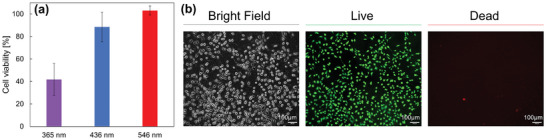
a) Cellular phototoxicity test for MDCK. After 24 h of MDCK culturing on the polystyrene dish, UV light (365 nm, 10 mW cm^‐2^), blue light (436 nm, 8.9 mW cm^‐2^), and green light (546 nm, 6.6 mW cm^‐2^) were individually irradiated to the cells. Cell viability was evaluated by WST assay by defining the signal without photoirradiation as 100% viability. b) Bright field images and live/dead staining of MDCK 24 h cultured on the interface of collagen‐coated P(mAzoA‐*r*‐DMAAm) after irradiation of cells at green light in the conditions described above.

Finally, we investigated the biocompatibility of P(mAzoA‐*r*‐DMAAm)‐based cell scaffold materials. A dimethylforamide (DMF) pre‐gel solution, including monomers, initiators, and cross‐linkers, was polymerized on a glass substrate modified with 3‐(trimethoxysilyl)propyl methacrylate, to which a vinylidene group was introduced. After polymerization, the solvent was replaced with PBS to obtain the P(mAzoA‐*r*‐DMAAm) hydrogel. P(mAzoA‐*r*‐DMAAm) hydrogel interface was modified with collagen by using (sulfosuccinimidyl 6‐(4'‐azido‐2'‐nitrophenylamino)hexanoate), 1‐[[6‐[(4‐azido‐2‐nitrophenyl)amino]‐1‐oxohexyl]oxy]‐2,5‐dioxo‐3‐pyrrolidinesulfonic acid monosodium salt (sulfo‐SANPAH) to promote cell adhesion. MDCK were seeded on the P(mAzoA‐*r*‐DMAAm) hydrogel interface. For cellular phototoxicity test, MDCK were cultured on the 96 well dish and was individually exposed to UV light (365 nm, 10 mW cm^−2^), green light (546 nm, 6.6 mW cm^−2^), and blue light (436 nm, 8.9 mW cm^−2^) with the typical photo‐isomerization inducing energy needed for *trans*‐ to *cis*‐ isomerization of azobenzene, for *trans*‐ to *cis*‐ isomerization of mAzoA, and for *cis*‐ to *trans*‐ isomerization of both azobenzene and mAzoA, respectively. As shown in **Figure** **4**a, the cell viability, estimated from the WST assay, dropped to ≈40% after irradiation with 365 nm light, which is a commonly used wavelength and intensity for the *trans*‐to‐*cis* photoisomerization of azobenzene. In contrast, the cell viability after irradiation with blue and green light remained almost the same as that of the cells cultured on the unirradiated gel interface. Figure 4b shows the bright‐field and fluorescence images of the live/dead assay for the MDCK culture after 24 h on the interface of the P(mAzoA‐*r*‐DMAAm) hydrogel. It can be seen that the cells adhered and spread on the P(mAzoA‐*r*‐DMAAm) hydrogel interface, retaining high cell viability. These results indicated that the collagen‐coated P(mAzoA‐*r*‐DMAAm) hydrogel is an excellent biocompatible in vitro material that supports cell adhesion. We also confirmed that the photoisomerization reaction of mAzoA in response to visible light successfully proceeded in the hydrogel within 30 min (Figure S11, Supporting Information). Furthermore, the light irradiation required for photoisomerization is nontoxic and does not threaten cell viability. We have shown that it is possible to construct cell scaffolds that can switch between different mechanical and/or chemical states under mild conditions compared to conventional azobenzene‐containing soft materials.

## 3 Conclusion

In this paper, we reported the preparation of thermo‐ and light‐sensitive polymers based on the photoisomerization of a visible‐light‐responsive azobenzene with an electron‐donating methoxy group at the ortho‐position. P(mAzoA‐*r*‐DMAAm) exhibited an LCST‐type phase transition in PBS. It was revealed that *T*_c_ and Δ*T*_c_ were controllable not only by the amount of mAzoA unit introduced but also by its molecular weight. By precise tuning of mAzoA composition and molecular weight of P(mAzoA‐*r*‐DMAAm), we have achieved cyclic soluble‐insoluble changes by non‐cytotoxic visible light switching at 37 °C, typical mammalian cell culture temperature. For a preliminary trial of their application to cell scaffold materials and biomedical applications, we attempted cell culture on a P(mAzoA‐*r*‐DMAAm) hydrogel. Excellent adhesion and spreading of MDCK cells were observed at the interface of the P(mAzoA‐*r*‐DMAAm) hydrogel. Light irradiation at 436 and 546 nm, which are required for the photoisomerization of mAzoA, did not affect cell viability, demonstrating the biocompatibility of the material.

## Conflict of Interest

The authors declare no conflict of interest.

## Supporting information

Supporting Information

## Data Availability

The data that support the findings of this study are available from the corresponding author upon reasonable request.
