# Supplementary material for: Reversible Solubility Switching of a Polymer Triggered by Visible‐Light Responsive Azobenzene Photochromism with Negligible Thermal Relaxation
Source: Macromol Rapid Commun. 2024 Aug 8;45(22):2400419. doi: 10.1002/marc.202400419 (PMC11583355; doi:10.1002/marc.202400419)
Supplement: Supplementary file 1 — Supporting Information [file MARC-45-2400419-s001.docx]

***Supporting Information***

Reversible Solubility Switching of a Polymer Triggered by Visible-Light Responsive Azobenzene Photochromism with Negligible Thermal Relaxation

*Takeshi Ueki^a)b)^*^†^*, *Yuna Osaka^c)^* ^†^*, Kenta Homma^a),^*^‡^*, Shota Yamamoto^a)^, Aya Saruwatari^a)b)^, Hongxin Wang^a)^, Masao Kamimura^c)^, and Jun Nakanishi^a),c),d)^**

1. Research Center for Macromolecules and Biomaterials, National Institute of Materials and Science, 1-1 Namiki, Tsukuba, Ibaraki 305-0044 Japan
2. Graduate School of Life Science, Hokkaido University, Kita 10, Nishi 8, Kita-ku, Sapporo, Hokkaido 060-0810 Japan
3. Graduate School of Advanced Engineering, Tokyo University of Science, 6-3-1 Niijuku, Katsushika-ku, Tokyo 125-8585 Japan
4. Graduate School of Advanced Science and Engineering, Waseda University, 3-4-1 Okubo, Shinjuku-ku, Tokyo 169-8555 Japan

^†^These authors equally contributed this work.

^‡^Present address: Department of Applied Chemistry, Graduate School of Engineering, Osaka University, 2-1 Yamadaoka, Suita, Osaka, 565-0871 Japan, and Center for Future Innovation (CFi), Graduate School of Engineering, Osaka University, 2-1 Yamadaoka, Suita, Osaka, 565-0871 Japan

To whom correspondence should be addressed: [UEKI.Takeshi@nims.go.jp](mailto:UEKI.Takeshi@nims.go.jp) (T.U.) and [NAKANISHI.Jun@nims.go.jp](mailto:NAKANISHI.Jun@nims.go.jp) (J.N.)

**Materials and Methods**

**Materials and chemicals**

4-[2-(2,6-Dimethoxyphenyl)-diazenyl]-3,5-dimethoxyphenol (mAzo), acryloyl chloride, 3-(trimethoxysilyl)propyl methacrylate, and dimethyl sulfoxide-*d*_6_ (99.9 atom%D) were purchased from TCI. Triethylamine, dichloromethane, chloroform, ethyl acetate, hexane, 2,2'-azobis(isobutyronitrile) (AIBN), *N*,*N*-dimethylformamide (DMF), sodium chloride, potassium chloride, disodium hydrogen phosphate 12-hydrate, potassium dihydrogen phosphate, ethylene glycol dimethacrylate (EGDMA), and 0.25 w/v% trypsin 1 mmol L^-1^ EDTA 4Na solution with phenol red were purchased from Wako. (sulfosuccinimidyl 6-(4'-azido-2'-nitrophenylamino)hexanoate), 1-[[6-[(4-azido-2-nitrophenyl)amino]-1-oxohexyl]oxy]-2,5-dioxo-3-pyrrolidinesulfonic acid monosodium salt (sulfo-SANPAH) and LIVE/DEAD^TM^ viability/cytotoxicity kits were purchased from Thermo Fisher Scientific. MDCK cells were obtained from the RIKEN Cell Bank. Minimum essential Eagle’s medium (MEM) and collagen type Ⅰ (Corning) were purchased from Sigma-Aldrich. 4-(2-Hydroxyethyl)-1-piperazineethanesulfonic acid (HEPES), Heated-inactivated fetal bovine serum (FBS), penicillin-streptomycin mixed solution, 1% MEM-nonessential amino acids, 1% sodium pyruvate, and 1% L-glutamine were purchased from Nacalai. A WST-8 kit was purchased from DOJINDO. Dimethylacrylamide (DMAAm) was a gift from KJ Chemicals. AIBN was recrystallized from methanol prior to use. DMAAm and EGDMA were passed through an aluminum oxide column to remove the acidic polymerization inhibitors before use. All other chemical reagents were used as received, unless otherwise noted.


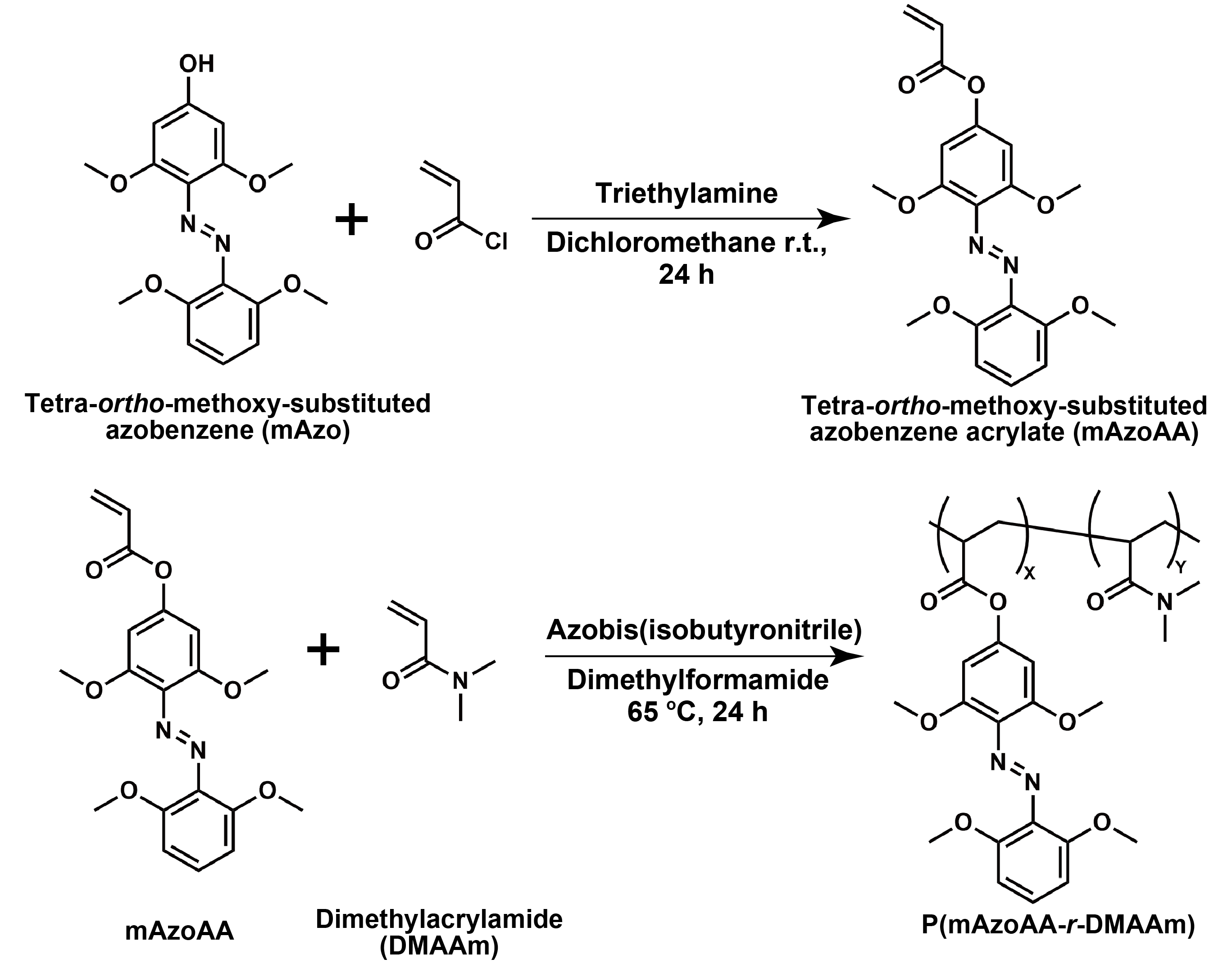


**Scheme S1.** Synthetic procedure of the mAzoAA monomer and P(mAzoAA-*r*-DMAAm).

**Synthesis of tetra-*ortho*-methoxy-substituted azobenzene acrylate (mAzoA) monomer**

First, 4.7 g of mAzo (0.015 mol) and 4.18 mL of triethylamine (0.030 mol) were dissolved in 90 mL of dichloromethane with stirring in a 300 mL two-necked round bottom flask. After argon purge, 60 mL of dichloromethane containing 3.64 mL of acryloyl chloride (0.045 mol) was added dropwise at 0°C cooling with an ice bath under vigorous stirring. The reaction was allowed to proceed overnight at room temperature. The crude crystals obtained by concentrating the filtrate were finally purified by silica gel column chromatography with a hexane : ethyl acetate = 2:1 mixture as an eluent, and then finally dried in vacuum at 80°C to obtain mAzoA monomer (**Scheme S1**). The chemical structure of mAzoA was characterized by ^1^H NMR (400 MHz, JEOL) (**Fig. S1**).

**Radical polymerization of P(mAzoA-*r*-DMAAm)**

P(mAzoA-*r*-DMAAm) was prepared by conventional radical polymerization of DMAAm with mAzoA. Herein, we describe a representative procedure for the synthesis of P(mAzoA_5.1_-*r*-DMAAm)_14.0kDa_ (**Scheme S1**). First, 111 mg of mAzoA (0.30 mmol) and 0.587 mL of DMAAm (5.70 mmol) was dissolved in 3 mL of DMF under stirring. A total of 3 mL of AIBN solution (0.030 mmol) was then added to the monomer solution and deoxygenated by purging with Ar for 10 min at room temperature. Free radical polymerization was carried out at 65°C for 24 h. After polymerization, the orange polymer solution was poured into a dialysis tube (molecular weight cutoff: 3.5 kDa, Thermo Fisher Scientific), which was then immersed in deionized water to remove unreacted monomers from the reaction solution. After dialysis, the polymer solution in the dialysis tube was freeze-dried (FDU-1110, EYELA) to obtain P(mAzoA-*r*-DMAAm). All the random copolymers were characterized using ^1^H NMR spectroscopy. **Figs. S2-S5** show ^1^H NMR spectra of P(mAzoA-*r*-DMAAm). The composition of mAzoA was calculated from the integrated intensity ratio between peaks sum of (a) and (a)' from *trans*- and *cis*-mAzoA, respectively, and (e) from DMAAm. The number average molecular weight (*M*_n_) and the polydispersity index (PDI: *M*_w_/*M*_n_) of the P(mAzoA-*r*-DMAAm)s were determined by size exclusion chromatography (SEC) using DMF containing 0.01 mol L^-1^ LiBr as the carrier solvent. The columns (SB-806M HQ, Showa Denko) used for SEC were calibrated using poly(methyl methacrylate) standards. The characterization results for all P(mAzoA-*r*-DMAAm) samples are summarized in **Table S1**. The mAzoA content incorporated into the random copolymers ranged from 5.1 to 10.7 mol%. The copolymer composition curve of DMAAm ([M_1_]) with mAzoA ([M_2_]) allowed us to obtain a monomer reactivity ratio of *r*_1_ = 0.953 and *r*_2_ = 1.071 by the well-established Finemann-Ross analysis^[1]^. Since the product or *r*_1_ and *r*_2_ gives almost 1, the connectivity of DMAAm and mAzoA seems to be ideal random. However, the monomer reactivity ratios obtained here are roughly estimated from the relationship between the feed and actual (final) DMAAm/mAzoA ratios in the polymer from ^1^H NMR (**Table S1**). For more quantitative discussion, the monomer reactivity ratio might be estimated from the amount of the consumed monomer during the initial period of polymerization.

**Photochromism of P(mAzoA-*r*-DMAAm)**

The photochromism of P(mAzoA-*r*-DMAAm) in phosphate buffered saline (PBS) (pH 7.4) was investigated. The absorption spectra of P(mAzoA-*r*-DMAAm) were recorded using a UV-2600 (SHIMADZU) instrument under thermally regulated conditions. Photoirradiation was performed using a high-pressure mercury lamp USH-250SC (USHIO). The wavelength and intensity of the irradiated light (UV light: 365 nm, 10 mW cm^-2^, blue light: 436 nm, 6.6 mW cm^‑2^, and green light: 546 nm, 8.9 mW cm^-2^) were adjusted using color filters (Edmund Optics). A heat-absorbing filter was used to dissipate the heat generated by the mercury lamp.

**Temperature-dependent transmittance of P(mAzoA-*r*-DMAAm)**

The lower critical solution temperature (LCST)-type phase separation behavior of P(mAzoA-*r*-DMAAm) was evaluated by recording the transmittance data using UV-vis spectroscopy. P(mAzoA-*r*-DMAAm) was first dissolved in PBS to a concentration of 0.5 wt%. After adding the polymer solution to the measurement cell, the transmittance of the polymer solution was recorded at 700 nm as its temperature was raised by 1°C min^-1^ while stirring. The temperature of the polymer solution was simultaneously recorded during the experiments using a temperature logger (LR5021, Hioki). In the analysis of the *trans*-type, the polymer solution was irradiated under blue light (436 nm) for 30 min using a mercury lamp equipped with a color filter before recording the data. For the analysis of the *cis*-type, the polymer solution was irradiated under green light (546 nm) for 30 min before recording the data. While recording the transmittance data, the polymer solution was constantly irradiated with the corresponding light to minimize thermal relaxation. The phase separation temperature (cloud point, *T*_c_) was determined as the temperature at which 50% transmittance was observed.

**Photo-induced phase separation of P(mAzoA-*r*-DMAAm) recorded at 37°C**

The photo-induced phase separation of P(mAzoA_10.7_-*r*-DMAAm)_3.0kDa_ was explored by recording the transmittance data using a UV-vis spectrophotometer. P(mAzoA_10.7_-*r*-DMAAm)_3.0kDa_ was dissolved in PBS to a final concentration of 0.5 wt%. The transmittance of the polymer solution was recorded at 700 nm at 37°C. During the measurements, the photoisomerization state of mAzoA was controlled by irradiating the solution with blue (436 nm) or green (546 nm) light. The top of the measurement cell was irradiated with a mercury lamp equipped with a color filter.

**Preparation of P(mAzoA-*r*-DMAAm) hydrogels**

The P(mAzoA-*r*-DMAAm) hydrogels for cell culture were prepared using a previously reported procedure^[2]^. Cover glasses (10 mm × 10 mm, Matsunami) were washed with methanol and dried with a stream of nitrogen gas. The cover glasses were then cleaned in a UV-ozone cleaner (UV235, Filgen) for 1 h to obtain silanol groups. The cleaned cover glasses were immersed in a methanol solution mixed with 0.4 wt% 3-(trimethoxysilyl)propyl methacrylate for 1 h at 25°C, followed by washing with methanol and then drying with a stream of nitrogen gas.

The hydrophobic cover glasses were prepared via a silane-coupling reaction. After the same procedures of washing and UV-ozone irradiation of a 30 mm × 30 mm cover glass, the glass was immersed in a toluene solution mixed with 1 wt% trichlorooctadecylsilane for 1 h at room temperature. The cover glasses were washed with toluene and methanol and then dried with stream nitrogen.

P(mAzoA-*r*-DMAAm) hydrogels were prepared as thin-layer sheet gels via free-radical polymerization. First, 0.111 g of mAzoA (0.3 mmol), 587 μL of DMAAm (5.7 mmol), 60.7 μL of ethylene glycol dimethacrylate (EGDMA, 0.3 mmol), and 10 mg of AIBN (0.06 mmol) were dissolved in DMF to prepare a 3 mL solution in a glass vial. The vial containing the solution was purged with Ar for 20 min for deoxygenation. After Ar purging, the pre-gel solution was transferred to a glove box under an Ar atmosphere. A 50 μL droplet of the pre-gel solution was cast on a hydrophobic substrate. The methacrylated cover glass was carefully placed on top of the droplet, with the methacrylated side facing down so that covalent bonds were formed between the cover glass and the gel. The gelation reaction was performed on the hot plate at 65°C in the Ar atmospheric glove box for 24 h. After the reaction, the sheet gels were immersed in methanol for 2 d to remove the unreacted monomers. The gels were immersed in PBS to exchange the solvent. The sheet gels on the cover glasses were subsequently immersed in HEPES buffer solution (50 mM, pH 8.5) containing 1 mg mL^-1^ sulfo-SANPAH. UV light was irradiated (UV crosslinker CL-1000L) on the sheet gels until an accumulated intensity of 10 J cm^-2^ was reached. The sheet gels were washed twice with PBS, immersed in a HEPES buffer solution containing 0.1 mg mL^-1^ collagen type Ⅰ, and incubated at 4°C overnight. The collagen-coated gels were washed twice with PBS and stored at 4°C until further use.

**MDCK cell culturing**

MDCK cells were cultured in Eagle’s minimal essential medium containing 10% heat-inactivated fetal bovine serum, 100 units mL^-1^ penicillin and 100 mg mL^-1^ streptomycin, 1% MEM-nonessential amino acids, 1% sodium pyruvate, and 1% L-glutamine in a humidified atmosphere at 37 ˚C with 5% CO_2_. Cell passages were performed as follows. The medium in the dish in which the cells were cultured was removed with an aspirator and washed with PBS; after the PBS was removed, 2 mL of 0.25 w/v% Trypsin-1 mmol L^-1^ EDTA solution was added and incubated for 5 minutes. The suspension was collected in a 15 mL tube to which 10 mL of medium was added and spun down in a small tabletop centrifuge (120 g, 2 min). After removal of the supernatant solution, 1 mL of medium was added and pipetted. A 100 μL of the cell suspension and 10 mL of medium were added to a 10 cm dish for cell passaging. Passages were performed every 2 or 3 days.

**Photo-toxicity test**

Photo-toxicity studies on MDCK cells were performed using WST-8. MDCK cells were seeded on 96 wells dish at a density of 5.0 × 10^3^ cells/well and incubated for 24 hours (5 % CO_2_, 37 ˚C). The medium was removed and replaced with medium containing 10% FBS in HBSS before irradiating the cells with light. The cells were irradiated with ultraviolet light (10 mW cm^-2^) for 1 hour, 436 nm light (8.9 mW cm^-2^) for 1 hour, 546 nm light (6.6 mW cm^-2^) for 30 minutes, and no light irradiation as a reference was used as dark. Light irradiation was performed on a 96 well plate with a polystyrene cover for visible light irradiation and a quartz cover glass for ultraviolet light irradiation. After irradiation, the plates were incubated for another 24 hours (5% CO_2_, 37 ˚C). Then, 10 μL of WST-8 was added to 100 μL of culture medium in each well, and after 2 hours of incubation (5% CO_2_, 37 ˚C), absorbance was measured at 450 nm using a microplate reader.


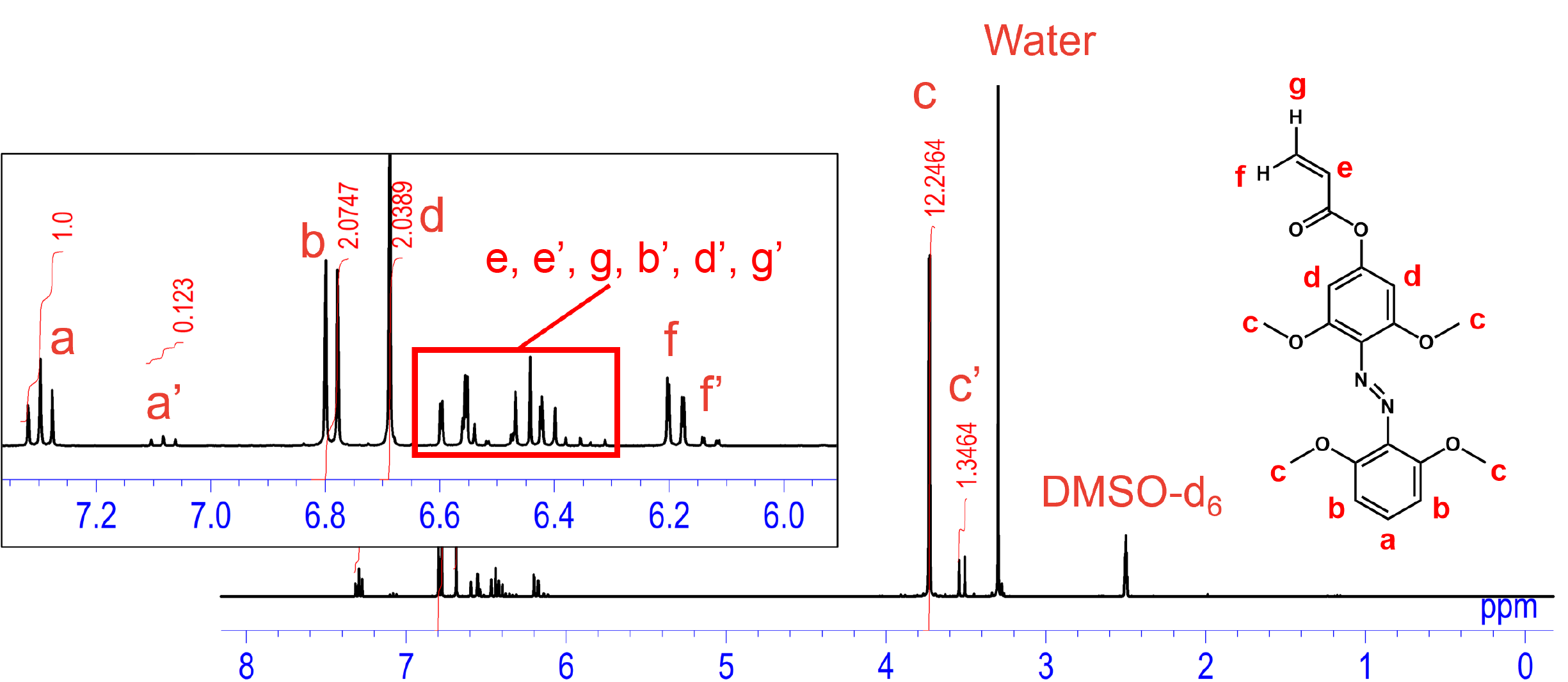


**Figure S1** ^1^H NMR spectrum of mAzoA monomer in dimethyl sulfoxide (DMSO)-*d*_6_. Each proton is assigned to the position shown in the chemical structure in the figure. The unprimed and primed protons are derived from the *trans*- and *cis*- photo isomerized species, respectively.


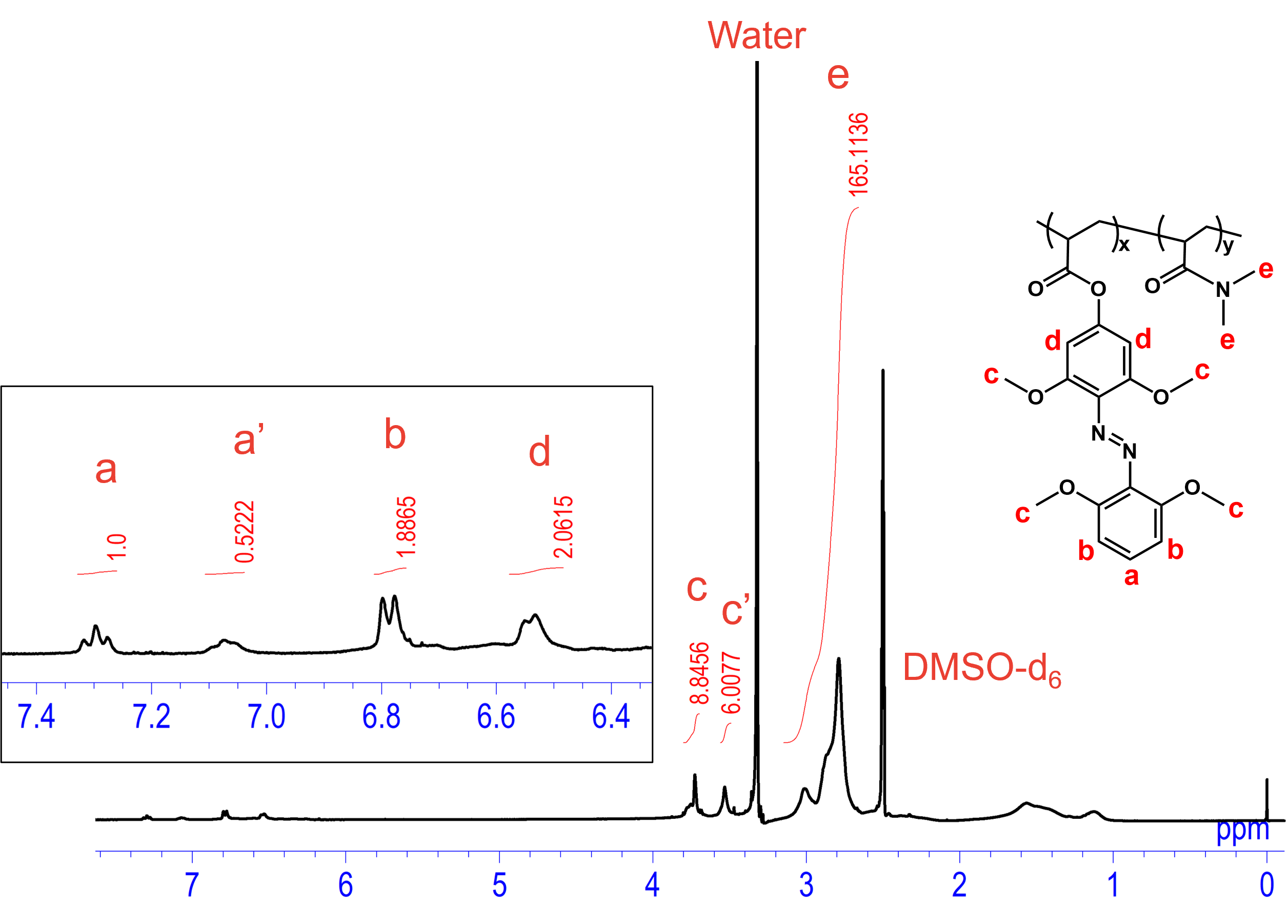


**Figure S2** ^1^H NMR spectrum of P(mAzoA_5.1_-*r*-DMAAm)_14.0kDa_ in DMSO-*d*_6_. Each proton is assigned to the position shown in the chemical structure in the figure. The unprimed and primed protons are derived from the *trans*- and *cis*- photo isomerized species, respectively.


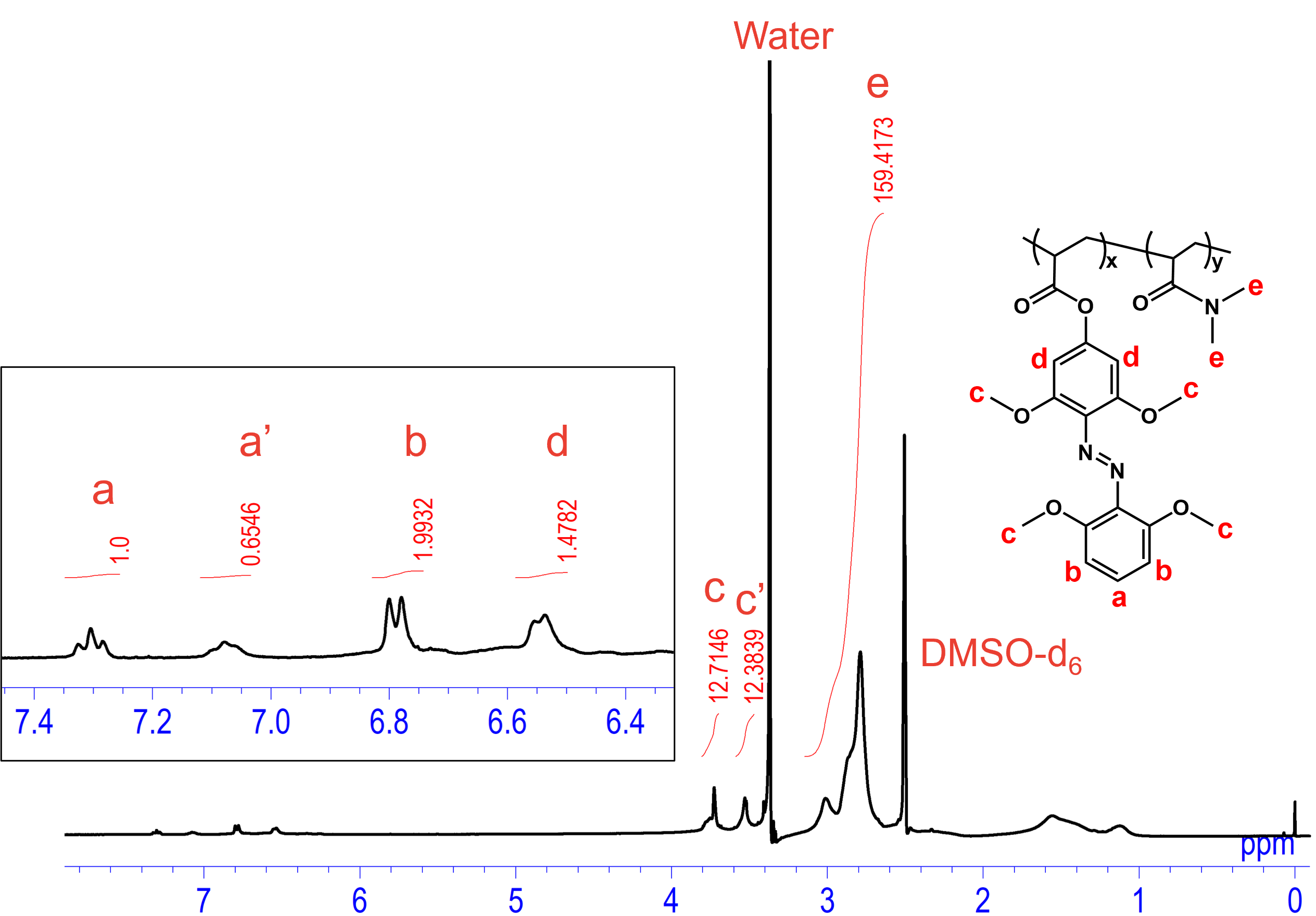


**Figure S3** ^1^H NMR spectrum of P(mAzoA_5.8_-*r*-DMAAm)_13.0kDa_ in DMSO-*d*_6_. Each proton is assigned to the position shown in the chemical structure in the figure. The unprimed and primed protons are derived from the *trans*- and *cis*- photo isomerized species, respectively.


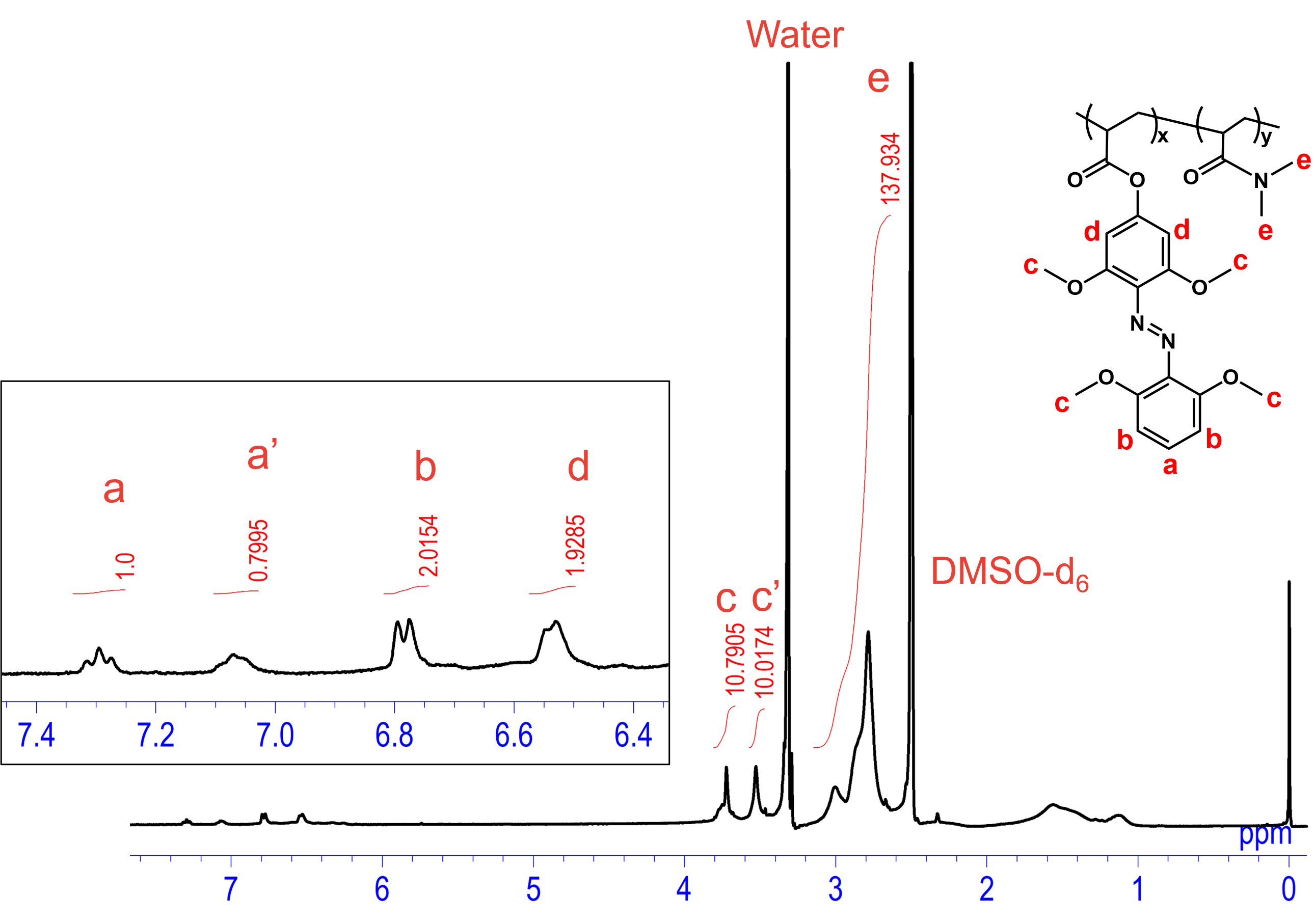


**Figure S4** ^1^H NMR spectrum of P(mAzoA_7.2_-*r*-DMAAm)_10.0kDa_ in DMSO-*d*_6_. Each proton is assigned to the position shown in the chemical structure in the figure. The unprimed and primed protons are derived from the *trans*- and *cis*- photo isomerized species, respectively.


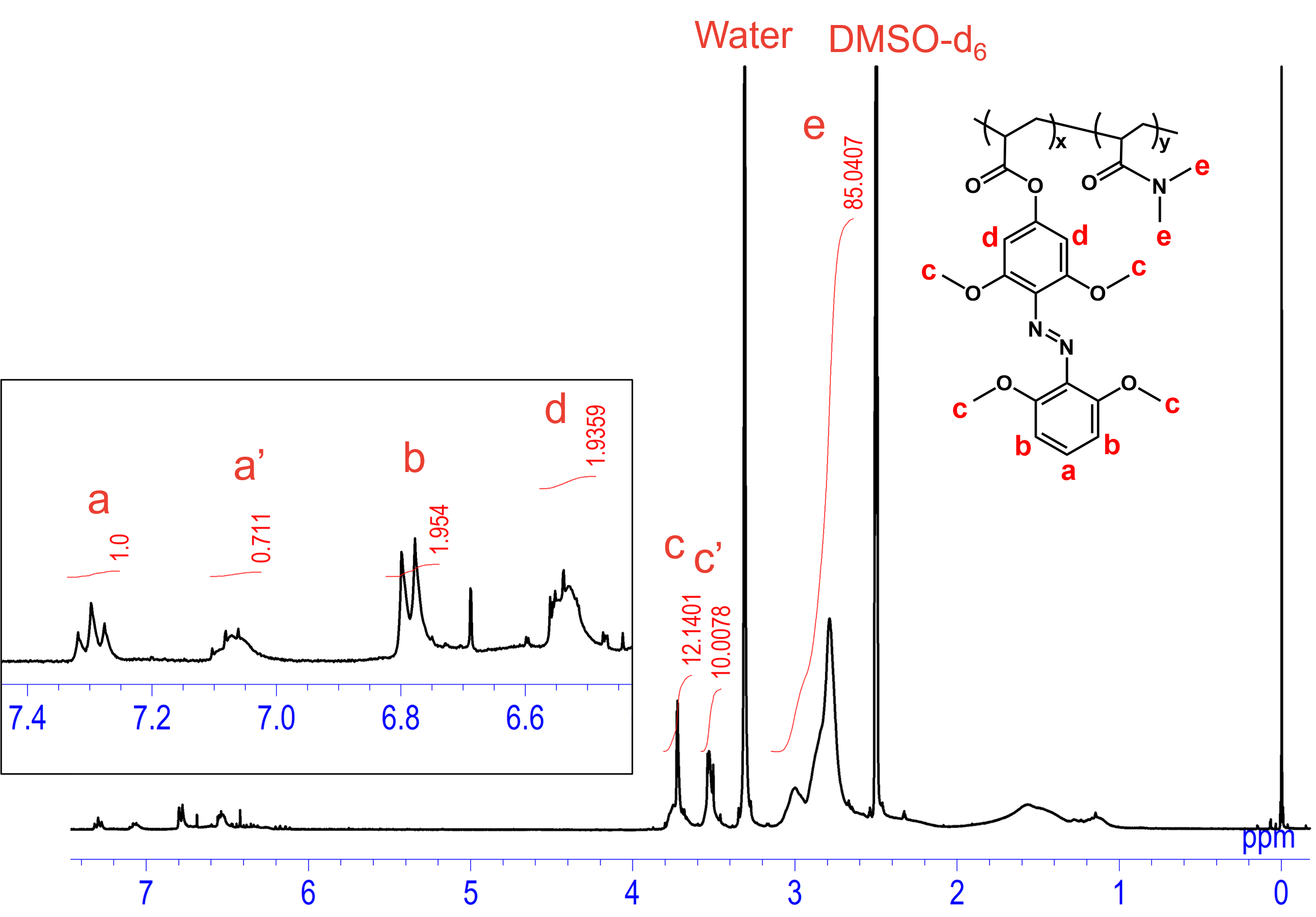


**Figure S5** ^1^H NMR spectrum of P(mAzoA_10.0_-*r*-DMAAm)_3.0kDa_ in DMSO-*d*_6_. Each proton is assigned to the position shown in the chemical structure in the figure. The unprimed and primed protons are derived from the *trans*- and *cis*- photo isomerized species, respectively.

**Table S1.** Summary of the characterization of P(mAzoA-*r*-DMAAm) used in this study.

|  | mAzoA composition / mol% | *M*_n_ / kDa | *M*_w_ / kDa | PDI (*M*_w_/*M*_n_) |
| --- | --- | --- | --- | --- |
| P(mAzoA_5.3_-*r*-DMAAm)_14kDa_ | 5.3 | 14 | 36 | 2.6 |
| P(mAzoA_6.2_-*r*-DMAAm)_13kDa_ | 6.2 | 13 | 35 | 2.2 |
| P(mAzoA_7.2_-*r*-DMAAm)_10kDa_ | 7.2 | 10 | 24 | 1.9 |
| P(mAzoA_10.7_-*r*-DMAAm)_3kDa_ | 10.7 | 3.0 | 5.0 | 1.5 |


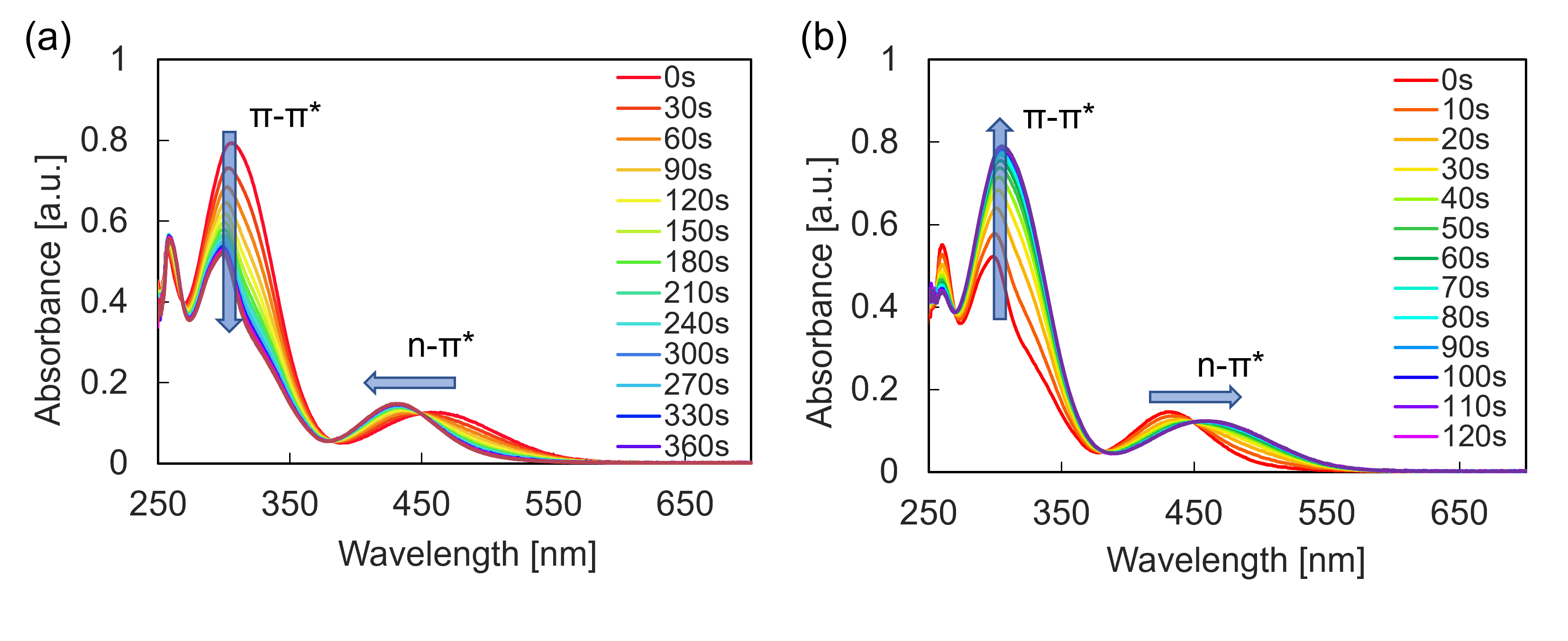


**Figure S6** UV-vis spectra of mAzoA monomer (0.03 w/v%) in DMSO-*d*_6_ of (a) photoisomerization process from *trans*- to *cis*-form under 546 nm irradiation and (b) photoisomerization process from *cis*- to *trans*-form under 436 nm irradiation.


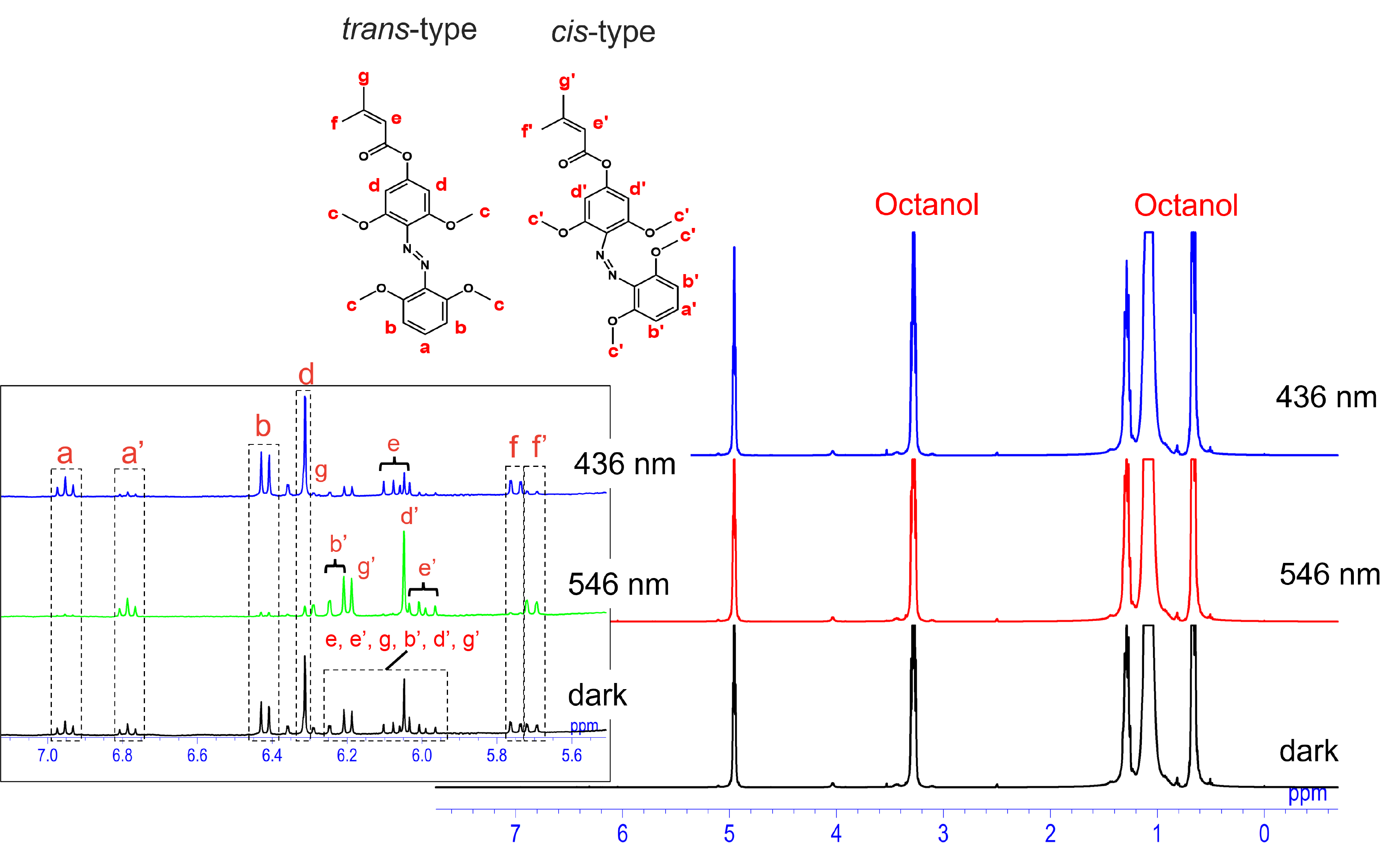


**Figure S7** ^1^H NMR of photoisomerization state of mAzoA monomer in octanol (blue) under 436 nm irradiation, (red) under 546 nm irradiation, and (black) under dark. Each proton is assigned to the position shown in the chemical structure in the figure. The unprimed and primed protons are derived from the *trans*- and *cis*- photoisomerized species, respectively.


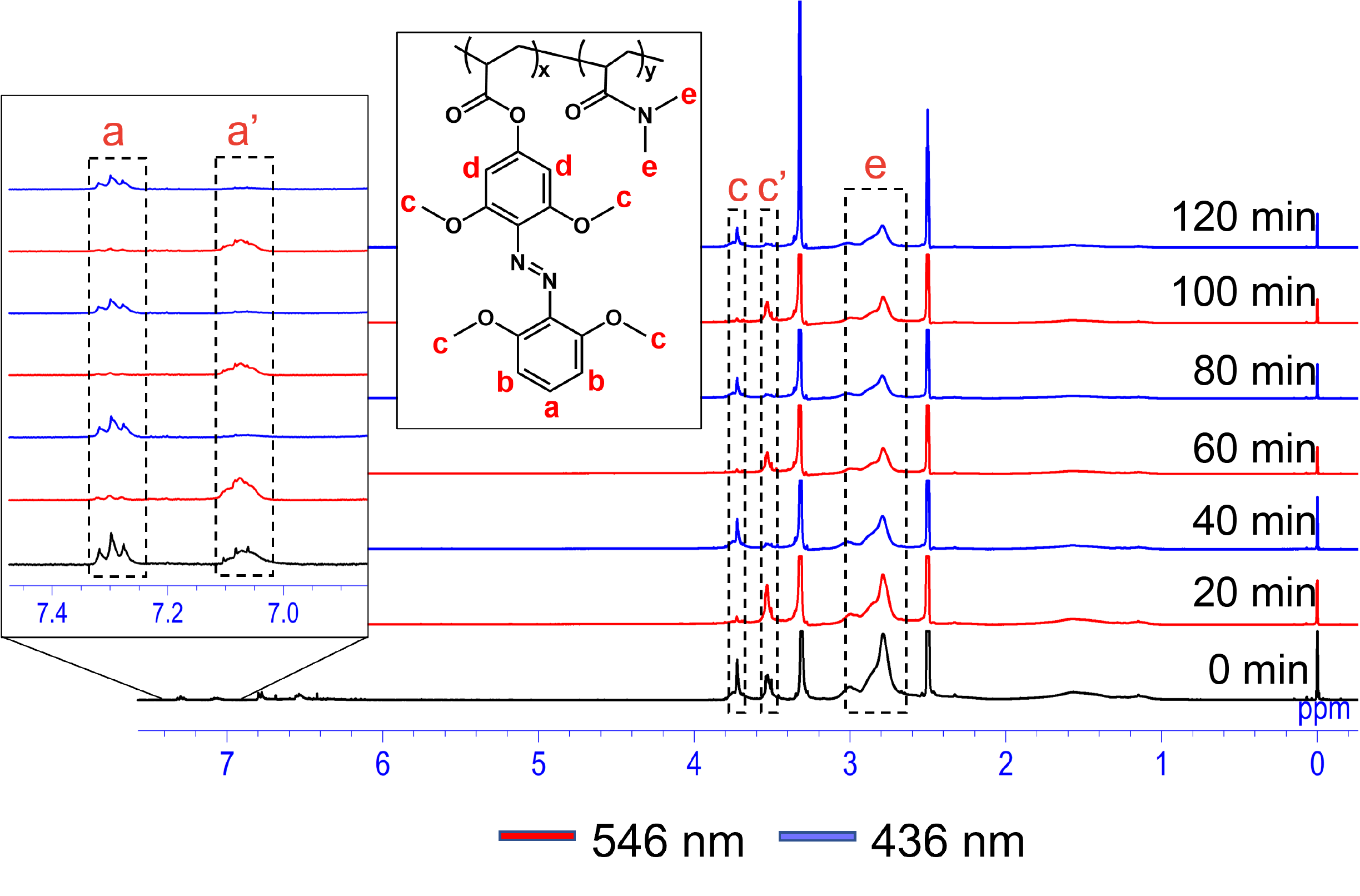


**Figure S8** ^1^H NMR spectra of P(mAzoA_10.7_-*r*-DMAAm)_3.0kDa_ in DMSO-*d*_6_ showing reversible photoisomerization state induced by alternative switching of irradiation light wavelength. The sample solution was alternatively exposed to green light (546 nm) and blue light (436 nm) in every 20 minutes. The unprimed and primed protons are derived from *trans*- and *cis*-photoisomerized species, respectively.


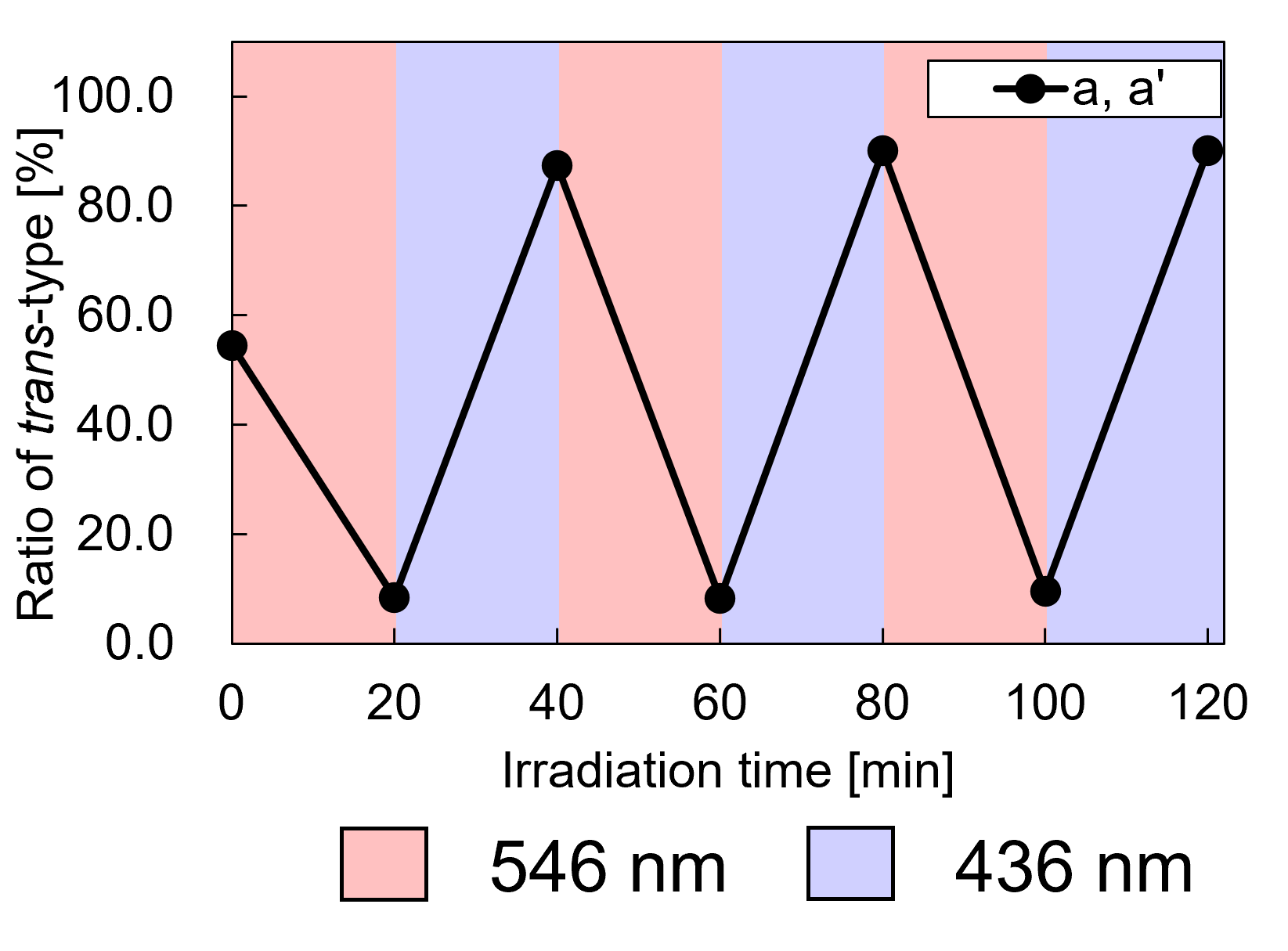


**Figure S9** Cyclic changes of ratio of P(*trans*-mAzoA_10.7_-*r*-DMAAm)_3.0kDa_ in the polymer solution. The ratio of P(*trans*-mAzoA_10.7_-*r*-DMAAm)_3.0kDa_ was determined by the integrated peak of proton “a” derived from *trans*-type and “a'” derived from *cis*-type appeared between 7.2 ppm and 7.4 ppm of **Figure S8**.


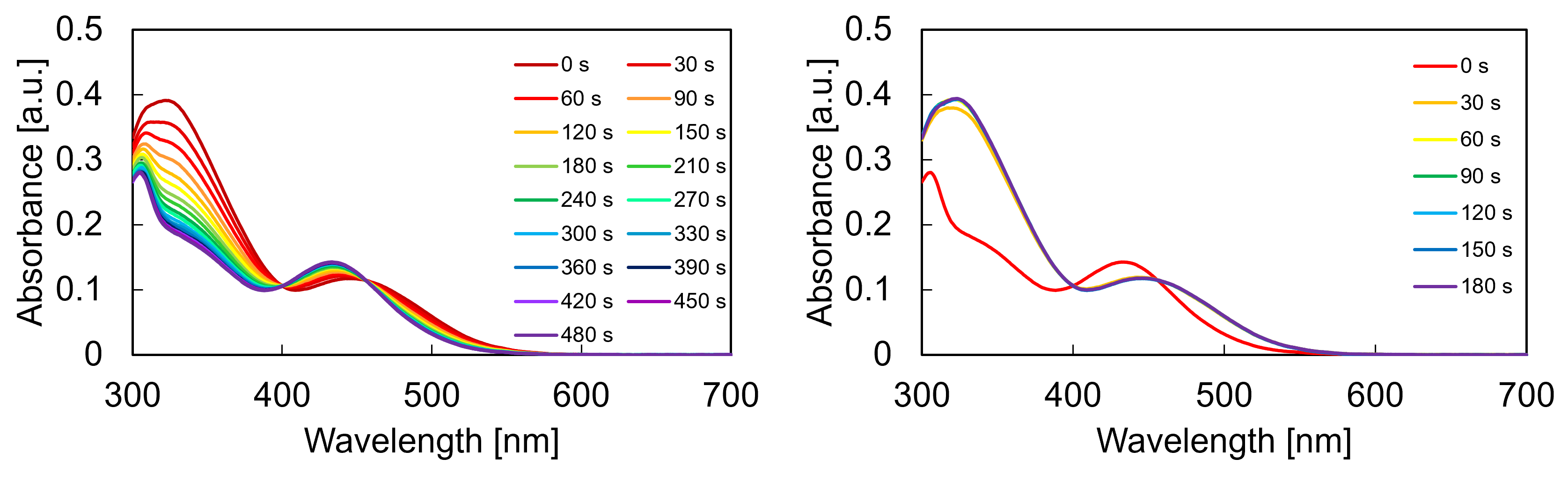


**Figure S10** UV-vis spectra of P(mAzoA_5.8_-*r*-DMAAm)_13.0kDa_ (0.01 w/v%) in PBS solution of (a) photoisomerization process from *trans*- to *cis*-form under 546 nm irradiation and (b) photoisomerization process from *cis*- to *trans*-form under 436 nm irradiation.

**Figure S11** UV-vis spectra of P(mAzoA-*r*-DMAAm) hydrogel irradiated with blue light (436 nm, 8.9 mW cm^-2^) and green light (546 nm, 6.6 mW cm^-2^) for 30, 60, and 120 min. The hydrogel was sandwiched between two glass substrates equipped with 60 μm silicone spacer to define the thickness of the sample.

**Discussion of the effect of [mAzoA] and molecular weight on phase transition temperature**

To investigate the impact of molecular weight as well as photoisomerization reaction on the phase transition behaviors of P(mAzoA-*r*-DMAAm) with different molecular weights and mAzoA compositions (**Figs. S12(a)-(c)**). **Fig. S12(d)** plots the *T*_c_s of *trans*- and *cis*-P(mAzoA-*r*-DMAAm) against mAzoA composition. When we looked at the lower mAzoA composition range (5.3-7.2%), *T_c_* became lower as increasing the mAzoA composition irrespective of the photoisomerization state of mAzoA. Such trend is generally observed for azobenzene-containing polymers because mAzoA is hydrophobic and the increase in hydrophobic domains lowers *T_c_* in general. On the other hand, further increase in the mAzoA feed concentration up to 10.7%, *T_c_* increased both for the *cis-* and *trans-*state. This opposite response is essentially attributed to the fact that the phase transition temperature of P(mAzoA-*r*-DMAAm) is affected by the molecular weight of the polymer itself. Particularly the azobenzene moiety hampers the polymerization reaction due to the increase in the rate of the chain transfer reaction in the radical polymerization system, thereby limiting elongation of polymers, as the amount of mAzoA in the feed increased (**Table S1**). Several reports have already proven that the azobenzene pendant monomer acts as a chain transfer agent, resulting in a decrease in the molecular weight of the polymers when the azobenzene monomer in the feed increases^[3]^. There is also evidence that PDI becomes narrower with an increase in the mAzoA feed ratio, implying that the polymerization process in the presence of the mAzoA monomer proceeds similar to radical telomerization (**Table S1**). To highlight the relationship between *T_c_* of the *cis-* and *trans-*type, we defined the difference as Δ*T*_c_ and replotted against the mAzoA composition (**Fig. S12(e)**). Here, we found that P(mAzoA_5.1_-*r*-DMAAm)_14kDa_, which has the lowest mAzoA content, shows a Δ*T*_c_ of only 0.8°C. This is due to the low azobenzene content in the polymer chain, which prevents a change in the polarity contrast between the *trans*- and *cis*-types. When the loading amount of azobenzene was gradually increased in P(mAzoA_5.8_-*r*-DMAAm)_13kDa_, the Δ*T*_c_ increased because the polarity contrast was enhanced. However, in the case of P(mAzoA_7.2_-*r*-DMAAm)_10kDa_, in which mAzoA composition increased to 7.2 mol%, the molecular weight of the polymer was, in turn, reduced to approximately 10kDa, as the rate of chain transfer (polymerization inhibitory) effect increases due to increasing mAzoA in the polymerization system in the feed. As a result, the molecular weight enters the region where the order of *T*_c_s of *trans*- and *cis*-types is merged, and the Δ*T*_c_ becomes smaller again. So far, phase transition of polymerizable azobenzene^[4]^ or its derivative^[5]^ with DMAAm as the main monomer has been studied and such non-linear relationship between Δ*T*_c_ and azobenzene composition has also been reported. Based on this trend, we succeeded in increasing the bistable temperature while increasing the amount of mAzoA to 10 mol%. We also confirmed that the effect of molecular weight on Δ*T*_c_ seemed to show a stronger correlation than that of mAzoA composition on the polymer chain. Δ*T*_c_ monotonically increased with an increase in the molecular weight of P(mAzoA-*r*-DMAAm), with almost negligible dependence on the mAzoA composition (**Fig. S12(f)**). In previous cases of thermo-responsive polymers, in which functional monomers that switch polarity in response to external stimuli, such as azobenzene^[3c, 6]^ and redox changeable metal^[7]^, are introduced as sub-monomers, it has been thought that the bistable temperature is often dominated by the amount of polarity-variable functional group. However, the present study suggests that molecular weight has a certain effect on determining the bistable temperature. The present study was not able to examine the systematic incorporation of mAzoA content with a fixed molecular weight due to the chain transfer properties of mAzoA comonomer itself. Therefore, the apparent correlation between molecular weight and *T*_c_ may need to be investigated more carefully in the forthcoming study. In addition to the molecular and mAzoA content, the effect of the polymer concentration on the responsive behavior should also be systematically investigated. Also, it is possible that the terminal group affects phase behavior especially in the case of low molecular weight P(mAzoA_10.7_-*r*-DMAAm)_3.0kDa_. Because when the molecular weight decreases, the effect of terminal groups on the phase transition reveals relatively greater. Particularly, since mAzoA of aromatic compound, which is reported to decrease LCST strongly^[8]^, acts as a chain transfer agent. It is probable that mAzoA group is introduced in the terminal polymer chain. The counterintuitive phase behavior observed in P(mAzoA_10.7_-*r*-DMAAm)_3.0kDa_ might be, for example, *trans*-type polymer form more thermally stable micelle like structure than individually dissolved *cis*-type polymer. Although clarification of the molecular mechanism of the molecular weight in the bistable temperature region remains rudimentary, this study implies that the molecular weight of stimuli-responsive polymers shall not be overlooked.


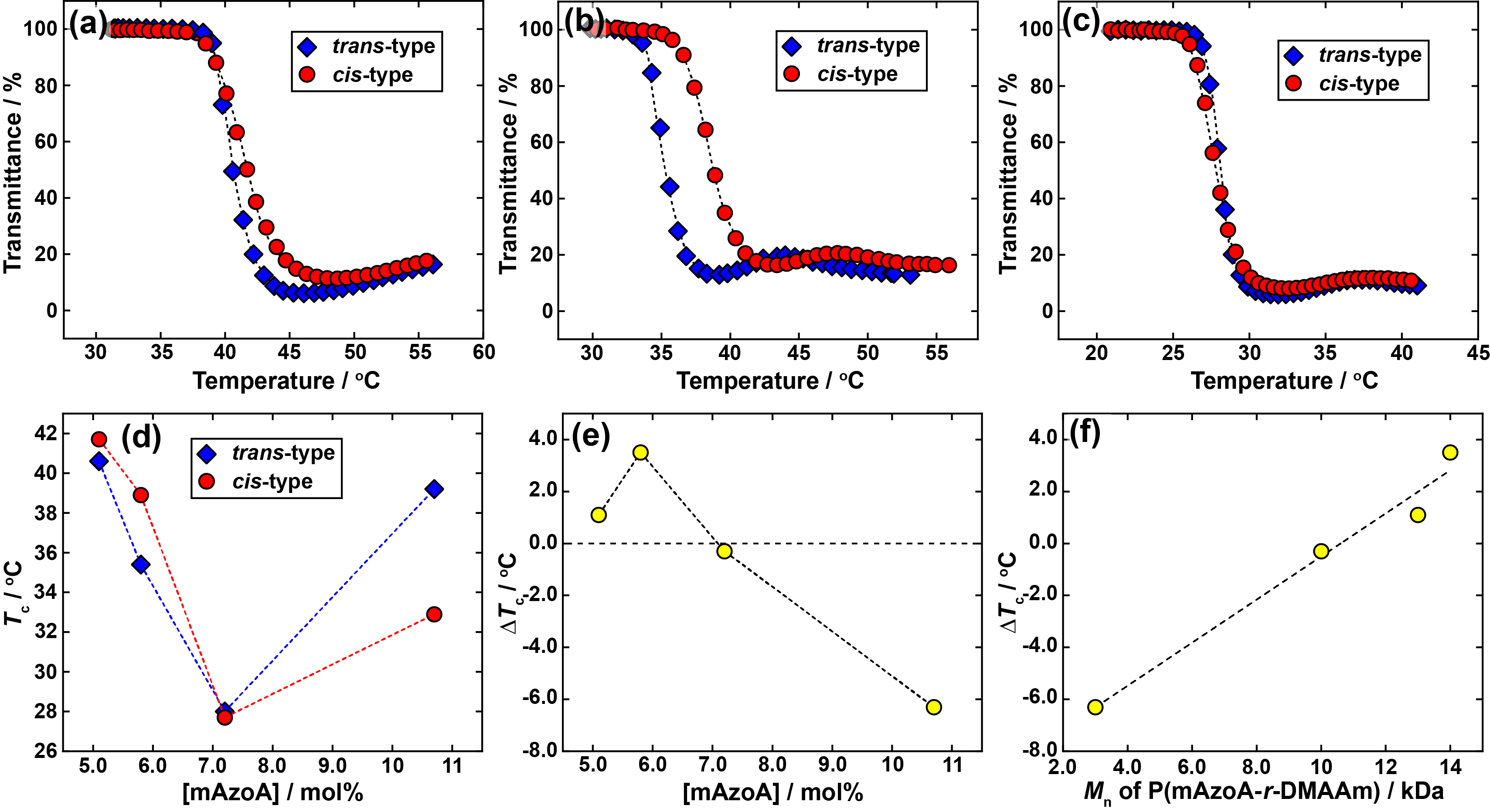


**Figure S12.** Temperature dependence of transmittance at 700 nm for (a) P(mAzoA_5.1_-*r*-DMAAm)_14kDa_, (b) P(mAzoA_5.8_-*r*-DMAAm)_13kDa_, and (c) P(mAzoA_7.2_-*r*-DMAAm)_10kDa_ in PBS (pH 7.4) solution. [Polymer] = 0.5 wt%. Scan rate: 1 °C min^-1^. Blue diamond plots and red circle plots indicate photoisomerization state of polymer as *trans*- and *cis*-type, respectively. (d) Relationship between *T*_c_ and the composition of mAzoA in P(mAzoA-*r*-DMAAm). *T*_c_ is defined as the temperature where the transmittance becomes 50%. (e) Relationship between Δ*T*_c_ and the composition of mAzoA in P(mAzoA-*r*-DMAAm). Δ*T*_c_ is defined as the difference between *T*_c_ of P(*cis*-mAzoA-*r*-DMAAm) and that of P(*trans*-mAzoA-*r*-DMAAm). (f) Relationship between Δ*T*_c_ and number average molecular weight (*M*_n_) of P(mAzoA-*r*-DMAAm).

**References**

[1] P. C. Hiemenz, T. P. Lodge, *Polymer Chemistry*, CRC Press, 6000 Broken Sound Parkway NW, Suite 300, Boca Raton, FL 33487-2742 **2007**.

[2] K. Homma, A. C. Chang, S. Yamamoto, R. Tamate, T. Ueki, J. Nakanishi, *Acta Biomater* **2021**.

[3] a)T. Ueki, Y. Nakamura, A. Yamaguchi, K. Niitsuma, T. P. Lodge, M. Watanabe, *Macromolecules* **2011**, 44, 6908; b)T. Ueki, Y. Nakamura, T. P. Lodge, M. Watanabe, *Macromolecules* **2012**, 45, 7566; c)H. Akiyama, N. Tamaoki, *J. Polym. Sci. Part A: Polym. Chem.* **2004**, 42, 5200.

[4] a)S. Deshmukh, L. Bromberg, K. A. Smith, T. A. Hatton, *Langmuir* **2009**, 25, 3459; b)F. D. Jochum, P. Theato, *Polymer* **2009**, 50, 3079.

[5] R. Steinbrecher, P. Zhang, C. M. Papadakis, P. Muller-Buschbaum, A. Taubert, A. Laschewsky, *Chem Commun (Camb)* **2024**, 60, 1747.

[6] a)T. Ueki, A. Yamaguchi, N. Ito, K. Kodama, J. Sakamoto, K. Ueno, H. Kokubo, M. Watanabe, *Langmuir* **2009**, 25, 8845; b)K. Sugiyama, K. Sono, *J. Appl. Polym. Sci.* **2001**, 81, 3056.

[7] T. Ueki, M. Onoda, R. Tamate, M. Shibayama, R. Yoshida, *Chaos* **2015**, 25, 064605.

[8] a)P. Kujawa, F. Segui, S. Shaban, C. Diab, Y. Okada, F. Tanaka, M. W. Francisco, *Macromolecules* **2006**, 39, 341; b)P. J. Roth, F. D. Jochum, F. R. Forst, R. Zentel, P. Theato, *Macromolecules* **2010**, 43, 4638; c)X. Qiu, T. Koga, F. Tanaka, F. M. Winnik, *Science China Chemistry* **2012**, 56, 56; d)H. Akiyama, N. Tamaoki, *Macromolecules* **2007**, 40, 5129.
